# Co-Infection by *Leptospira montravelensis* and *Leptospira interrogans* Serovar Pomona in Urine Samples of Donkeys and Pigs in Sardinia, Italy

**DOI:** 10.3390/ani13111803

**Published:** 2023-05-30

**Authors:** Ivana Piredda, Loris Bertoldi, Aureliana Pedditzi, Pierangela Pintore, Bruna Palmas, Valentina Chisu

**Affiliations:** 1 Laboratory of Sieroimmunology, Animal Health Department, Istituto Zooprofilattico Sperimentale della Sardegna, 07100 Sassari, Italy; aureliana.pedditzi@izs-sardegna.it (A.P.); pierangela.pintore@izs-sardegna.it (P.P.); bruna.palmas@izs-sardegna.it (B.P.); valentina.chisu@izs-sardegna.it (V.C.); 2 BMR Genomics s.r.l., Via Redipuglia 22, 35131 Padova, Italy; loris.bertoldi@bmr-genomics.it

**Keywords:** donkey, *Leptospira*, leptospirosis, mammalian, *montravelensis*, pig, saprophytic

## Abstract

**Simple Summary:**

Leptospirosis is one of the most neglected zoonotic diseases in the world and humans can easily acquire the pathogen after exposure to water, soil, or mud contaminated with urine from infected animals. In September 2018, a leptospirosis outbreak caused by a pathogenic *Leptospira* genospecies *interrogans* was identified in one farm in North Sardinia, Italy. In addition, culture and isolation of a saprophytic *Leptospira* genospecies *montravelensis* from two urine samples (from one pig and one donkey) was reported for the first time, indicating that co-infection with more than one strain of *Leptospira* in the same reservoir host is possible. These results provide new information on the epidemiology of leptospirosis and on the genotypes circulating in Sardinia, emphasizing the conducting of active surveillance of leptospirosis in humans and animals.

**Abstract:**

Saprophytic leptospires are spirochetes enclosed within the non-pathogenic clade of the genus *Leptospira,* which in turn is subdivided into two subclades S1 and S2. To date, the microorganisms included in these subclades have been isolated from the environment in various parts of the world, and are believed to have no known animal reservoirs. After a case of *Leptospira interrogans* serovar Pomona was notified to the owner of a farm in Sardinia, all of the farm animals (11 pigs and 3 donkeys) were examined for the presence of *Leptospira*. Sera of all tested animals resulted positive for antibodies to *Leptospira* using a microscopic agglutination test (MAT). Moreover, nine (82%) kidney samples from pigs and three urine samples collected from donkeys (100%) tested positive for *Leptospira* DNA after qPCR. Results obtained after MLST analysis and sequencing of *rrs*, *rpoB*, and *secY* genes, performed on six *Leptospira* strains isolated in culture, revealed the presence of the genomospecies *L. interrogans* serovar Pomona in the kidney samples. Conversely, whole-genome sequencing combined with mean nucleotide identity revealed the presence of the saprophytic *L. montravelensis* in the urine samples. Our results report, for the first time, the isolation of a saprophytic species from mammalian urine, suggesting a new ecological specialization for these bacteria, with a possible transition from free-living to a symbiotic lifestyle. Further studies will have to be conducted to understand the evolution of virulence of these bacteria, potential infectivity, and possible public health implications.

## 1. Introduction

Leptospirosis is a cosmopolitan bacterial zoonosis classified by the World Organization for Animal Health as a reemerging infectious disease [1,2,3]. Animals can contract the pathogen when they are in contact with infected animals or a contaminated environment [4]. Maintenance hosts generally do not develop clinical forms of the disease, but act as natural sources of pathogens, strongly influencing the epidemiology [5]. Due to the ability of leptospires to survive in soil and water for long periods, the host susceptibility range of *Leptospira* species is extremely wide [6,7]. Large outbreaks of leptospirosis have been reported worldwide, mainly in countries with tropical or subtropical climates, and in resource-poor countries, where, due to the absence of effective vaccines, treatments, and adequate sanitation practices, the eradication of the disease represents a major challenge [8,9,10]. Furthermore, since the spectrum of mammalian hosts harboring and excreting the different *Leptospira* species from their renal tubules is broad and animals are often asymptomatic, the burden of leptospirosis has been underestimated and the ability to prevent infection and control animal diseases is lacking [11,12,13].

Recently, *Leptospira* species have been classified into four subclades, P1, P2, S1, and S2, based on genome sequences [14]. The 38 pathogenic species belonging to P1 and P2 can cause leptospirosis, while the 26 saprophytic species included in subclades S1 and S2 have been isolated in a wide variety of environments including surface water and soil [15]. Leptospirosis is a disease that affects certain categories of at-risk workers exposed to animals that act as reservoirs or contaminated environments, such as slaughterhouses, wastewater workers, and individuals participating in water sports and recreational activities.

In Italy, leptospirosis is a compulsory-notification disease where, in the case of an outbreak, several measures are taken to control the rapid spread of the disease, including a strict policy that includes establishing quarantine zones for the isolation of infected animals from healthy ones and stringent culling of fetuses and fetal wrappings (when it is not possible to send them urgently and in safe conditions, also for the purposes of differential diagnosis, to the laboratories referred to in art. 67 of the veterinary police regulation).

In Sardinia, the incidence of *Leptospira* in animals is often reported, and new cases of leptospirosis caused by different pathogenic and intermediate species of the bacterium have been registered in vertebrate hosts on the island over the past five years [16,17,18]. Leptospirosis is well-recognized as a major global cause of reproductive failure in pigs, with the Tarassovi [19], Pomona [20], and Australis [21] serogroups predominating. Incidental infections in pigs may be associated with hemorrhagic disease, hematuria, jaundice, and acute kidney damage [22]. Farmers on the island are susceptible to the disease and know that eradication of the disease is financially and emotionally costly. These measures inevitably cause great economic losses and can affect many people and related industries.

In September 2018, after a case of human infection by *Leptospira interrogans* serovar Pomona, an investigation was carried out on pigs and donkeys living on one Sardinian farm owned by the infected man. With the aim of detecting *Leptospira* species with zoonotic potential, all animals from the farm were screened by combining bacterial isolation strategy, molecular diagnosis, and whole-genome sequencing (WGS). Herein, we report the first isolation and molecular typing of one saprophytic *Leptospira* strain from pig and donkey urine samples.

## 2. Materials and Methods

### 2.1. Sample Collection and MAT Analysis

In September 2018, after a confirmed case of *Leptospira* in a farmer, all animals from the farm were tested for the presence of *Leptospira* species. The property had 11 Sardinian pigs and 3 donkeys raised in a semi-wild management system. Prior to the visit, the farm was contacted by telephone and informed of the study in order to obtain informed consent. Strict biosecurity measures were also taken at every farm visit. Blood was collected in vacutainer tubes, placed on ice in an icebox, and then transported to Istituto Zooprofilattico Sperimentale della Sardegna (IZS), where samples were separated by centrifugation, aliquoted, and stored at −20 °C until further analyses. A microscopic agglutination test (MAT) was performed in accordance with standard procedure [23] using a panel of nine different serovars usually detected in Mediterranean area. Agglutination was examined by dark-field microscopy at a magnification of 100×. Titres > 1:200 were considered as evidence of previous exposure to *Leptospira*, according to Center for Disease Control and Prevention guidelines (https://wonder.cdc.gov/wonder/prevguid/topics.html accessed on 7 March 2023). After the results of the MAT, the farm was visited by a team of qualified veterinarians with professional experience in pig production. After notification of the presence of *Leptospira*, with the consent of the farm owner in agreement with the Prevention Department of the ATS Sardinia, all pigs were slaughtered and uncontaminated urine and kidney samples were collected. The collected urine samples by sterile cystocentesis from the bladder, were placed in a sterile tube containing *Leptospira* transport medium (LTM) and stored at −20 °C until use. Kidney samples were also collected from each dead animal. For this purpose, 25 mg of tissue extracted from the cortical and medullary areas were immediately used for culture. In addition, two water samples were taken from the pig and donkey troughs, respectively.

### 2.2. DNA Extraction and Molecular Detection of Leptospira spp. by Multiplex qPCR

Nucleic acid isolation was performed with the commercial DNeasy Blood and Tissue Kit^®^ (Qiagen, Hilden, Germany), according to the manufacturer’s instructions. Urine and kidney samples were analyzed for detection of *Leptospira* spp. by two real-time PCR (qPCR1) assays. In the first PCR assay, primers LipL32-45F (5′-AAG CAT TAC CGC TTG TGG TG-3′), LipL32-286R (5′-GAA CTCCCA TTT CAG CGA TT-3′), and the probeLipL32-189P (FAM-5′-AAA GCC AGG ACA AGC GCCG-3′-BHQ1) [24] were combined with primers 16S-P1 forward (5′-TAG TGA ACG GGA TTA GAT AC-3′) and 16S-P2 reverse (5′-GGT CTA CTT AAT CCG TTA GG-3′) and probe 16S-Prob (Cy5-5′-AAT CCA CGC CCT AAA CGT TGT CTAC-3′-BHQ2), which amplified 242 and 104 bp of the *lipL32* and *16S rRNA* genes, respectively. A second PCR assay (qPCR2) was carried out by using primers F1(5′-GAG TAA CAC GTG GGT AAT CTT CCT-3′), R3 (5′-TTT ACC CCA ACT AGC TAA TC-3′), and the probe Interm-16S (FAM-5′-GGA AAG CTA ATA CCG GAT AGT YCT RYT GGA-3′-BHQ1) [25], combined with primers 23S F (5′-ACA ATC TTA CCA AAC CCT ATC-3′), 23S R (5′-TTA CCA CTT AGC GTA GAT TT-3′), and the probe 23S P (JOE-5′-TCC GAA TAC TGT AAC TTG AAG TAC TGCA-3′-BHQ1) [26], which simultaneously amplify a highly conserved region of the *16S* gene specific for intermediate *Leptospira* species amplification and a highly selective region of the *23S* gene specific to saprophytic species. The reaction mix and PCR protocol were the same as used in a previous study [16]. *L. interrogans* ATCC BAA1198D5TM was used as positive control and included in each run. DNAse-free RNAse-free water was used as a negative control. If the Ct value was not detected, or it was ≥40, or a non-repeatable, the samples were considered negative.

### 2.3. Cultural Assay

The urine and kidney samples were cultured in semisolid Ellinghausen–McCullough–Johnson–Harris (EMJH)-enriched commercial medium (Difco, BD, Franklin Lakes, NJ, USA) supplemented with 5-fluorouracil (5-FU) (2 g/L), as previously described [16]. Cultures were incubated at 30 °C, and observed weekly under a dark-field microscope (40× objective). Samples that failed to show any evidence of growth after 3 months were considered negative and were discarded. When spirochetes were observed, positive liquid cultures were subjected to an additional step of purification that consisted of plating 100 µL of the diluted culture onto a solid EMJH agar (8 gL^−1^) as previously reported [27]. Once colonies were isolated and visible until the agar subsurface, three colonies from each plate were collected (according to their size, color, and opacity) by a needle of a sterile syringe and then sub-cloned into 9 tubes containing liquid EMJH medium. When the density reached 1 × 10^8^ leptospires, the genomic DNA (gDNA) was extracted from 200 µL of EMJH liquid clonal cultures in a final elution volume of 100 µL, and amplification of the *lipL32*, *16S*, and *23S* fragment genes was performed using primers set as above that allow the detection of pathogenic, intermediate, and saprophytic *Leptospira* species, respectively.

### 2.4. MLST Genotyping and Amplification of rrs, rpoB, and secY Genes

To reveal the sequence types (STs) of the *Leptospira* strains here isolated, Multilocus Sequence Types (MLST) assay was performed using the seven housekeeping genes as proposed by Boonsilp et al., 2013 [28]. Each allele and allelic profiles were submitted to the *Leptospira* database (http://pubmlst.org/leptospira, accessed on 23 February 2023) to define the STs. Furthermore, the isolates were analyzed with a primer series that amplified a 541 bp fragment of the *16S rRNA* gene, a 549 bp fragment of the partial *secY* gene [16], and a fragment of the (*rpoB*) RNA polymerase beta subunit gene [29]. One negative and one positive control were included in each analysis. PCR reactions were then performed using a T100 Thermal Cycler (Bio-Rad equipment) and amplification products were visualized by electrophoresis on 1.5% agarose gel stained with SYBR-Safe DNA Gel Stain (Invitrogen, Carlsbad, CA, USA), and examined under UV transillumination.

### 2.5. Whole-Genome Sequencing

In order to obtain DNA sequence information of *Leptospira* isolated from pigs and donkeys (strain 1079554), total DNA was sequenced using Illumina MiSeq technology (this Whole-Genome Shotgun project was deposited at DDBJ/ENA/GenBank under accession number JANLJQ0000000) [30]. The library was sequenced according to the v3 paired-end 300 bp read strategy, which produced 1.366.977 paired-end sequences. Pre-processing of the reads was first performed with FastQC v0.11.9 and the clean reads were then profiled using the ChocoPhlAn database [31] excluding possible contamination from other bacterial species. Lineage analysis revealed a complete overlap with the class *Spirochaetia* (239/239 BUSCO) [32], and this finding was also supported by MetaPhlAn [31], where the sample was associated with *L. montravelensis* strain (accession number NZ_ANIJ0000), without presenting any type of contamination.

## 3. Results

### 3.1. Sample Collection and MAT Results

A total of 14 blood samples obtained from 11 swine and 3 donkeys were tested by MAT analysis at the IZS Sardinia where all investigation was conducted.

All pigs and donkeys tested were defined as positive infected cases and showed titer antibody ≥1:800 for *L. interrogans* serovar Pomona in serum agglutination (MAT) according to the guidelines made by the “Leptospirosis Reference Epidemiology Group” (LERG) (Table 1).

After MAT diagnosis, the animals were slaughtered after stunning by electronarcosis, in accordance with Legislative Decree 333/1998 implementing Directive 93/119/EC, in the presence of an animal welfare officer to ensure that the animals showed no signs of consciousness or sensibility between the end of the stunning process and death.

### 3.2. Molecular Detection of Leptospira spp. from Urine and Kidney Samples

All urine and kidney samples collected from slaughtered pigs and urine from the three donkeys, showed leptospiral DNA after qPCR1 amplification (specific for the detection of all pathogenic species from *Leptospira* genus) using the lipL32 primers (Table 2). A second step of amplification was performed with primers as in qPCR2 specific for intermediate and saprophytic *Leptospira*. Results from this amplification analysis highlighted that only saprophytic species were detected from these samples.

**Figure 1:**
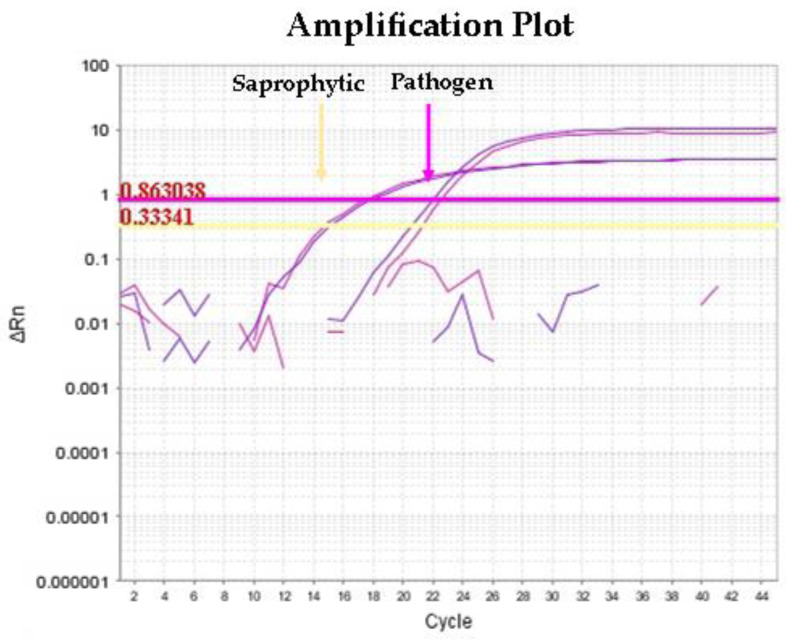
Amplification plot at multiplex qPCR shows simultaneous target amplification for pathogenic and saprophytic *Leptospira*, observing a higher concentration for the saprophytic species target. Yellow and pink string lines indicate the Ct Threshold belong to saprophytic and pathogen *Leptospira* strains, respectively.

When the qPCR2 was modified combining the primer specific for pathogen and saprophytic species (from now named qPCR2mod), the results clearly showed the presence of a double peak relative to the presence of both pathogen and saprophytic species in the urine samples of pig 5, pig 9, and pig 10 (Figure 1).

After 20 days in EMJH liquid medium, six cultures showed the motility and morphology typical of the genus *Leptospira* genus by dark-field microscopy.

Specifically, the frequency of positive urine cultures was 2/6 (33%), while that obtained from kidney cultures was 4/11 (36%), as presented in Table 2. Positive cultures from kidney samples were confirmed in pigs’ number 3, 4, 5, and 8 (all without clinical symptoms), and the results did not differ by sex or age, while positive urine cultures were confirmed in pig number 5 and donkey number 3.

### 3.3. Molecular Identification by PCR and MLST from Urine and Kidney Samples

MLST analysis performed on the six isolates using the seven housekeeping genes of scheme 1 [27] allowed us to identify one *Leptospira* Sequence type (ST) belonging to ST140 (derived from kidney samples of pig 3, 4, 5, and 8). However, no STs were obtained on isolates from the urine of pig 5 and donkey 3 after comparing the seven MLST loci database from the *Leptospira* MLST website (https://pubmlst.org/leptospira/ accessed on 9 April 2023), as reported in Table 3. In addition, the MLST sequences from kidney samples produced a clear chromatogram, unlike that obtained from urine samples. The six positive strains isolated from liquid EMJH medium were also analyzed by amplification of *rrs*, *rpoB*, and *secY* gene fragments and further sequencing using the same primers used for amplification. The results indicated that the sequence types of the strains isolated from kidney samples were identical to *L. interrogans* genomospecies (ST 140) as shown in Table 3.

Since the presence of saprophytic *Leptospira* was hypothesized after failed MLST analysis of the isolates from urine samples, qPCR2 was performed on the nine sub-cloned colonies obtained after the positive liquid cultures were plated on solid medium plates. The results obtained confirmed the presence of saprophytic species in these samples (Table 3). By retesting the same samples by qPCR2mod using the primers specific for pathogenic and saprophytic *Leptospira* species, the obtained amplification plot highlighted the coexistence of two *Leptospira* strains in the same urine samples, as in Figure 1. Intermediate *Leptospira* species were not found in the nine sub-cloned isolates. In addition, the qPCR2 results of the environmental samples gave information on the presence of one saprophytic *Leptospira* species that yielded different results from that obtained in urine samples.

### 3.4. Whole-Genome Sequencing of Leptospira Strains and Phylogenetic Analysis

**Figure 2:**
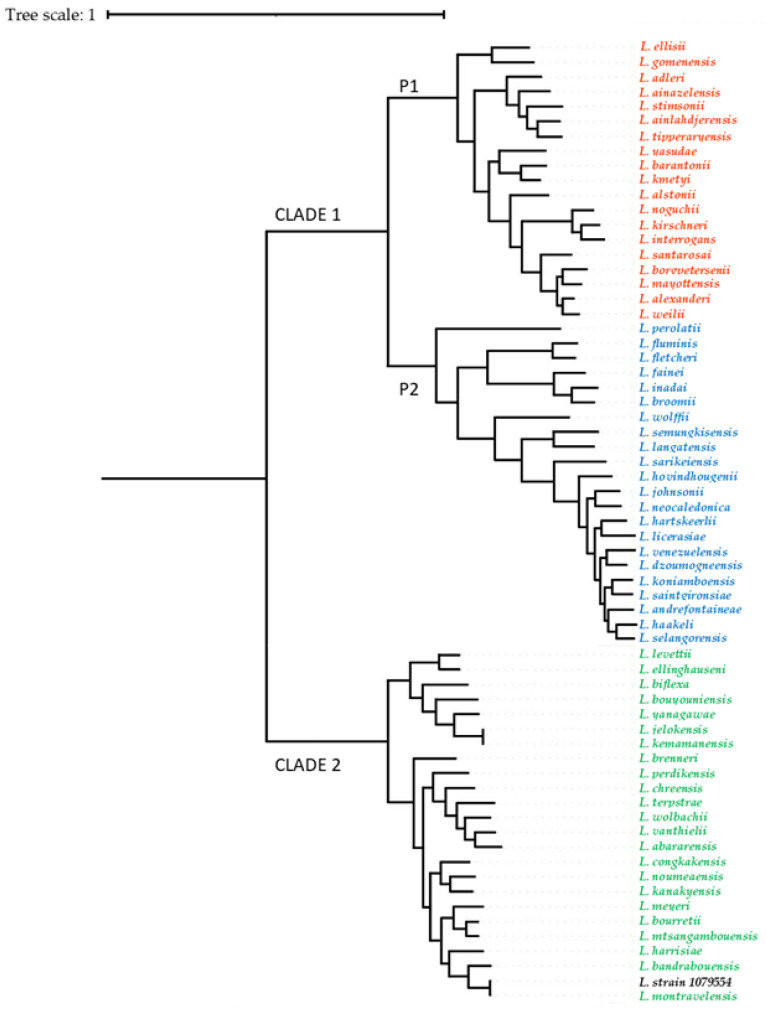
Phylogenetic tree of the *Leptospira* spp. generated in this study via PhyloPhlAn version3. The colors code the sequences belonging to the major group clades to which the different *Leptospira* species belong: red = pathogens; blue = intermediates or opportunists; green = nonpathogens.

The whole-genome sequencing obtained from saprophytic *Leptospira* strains isolated from two urine samples (from one donkey and one pig) are available in GenBank under accession number JANLJQ000000000 (BioProject accession number PRJNA867047, BioSample accession number SAMN30183847, and SRA accession number SRR22308640) as reported in Piredda et al., 2023 [30]. To determine definitively whether our two strains were closely related to the *L. montravelensis* species, we calculated the average nucleotide identity (ANI) [33,34] with JspeciesWS [35]. The mean nucleotide similarity between our isolated *Leptospira* strains and the *L. montravelensis* strain (GenBank accession number NZ_RQFN000000) was 99.30%. Phylogenetic analysis based on whole-genome sequencing of isolate 1079954 placed the sequence within the saprophytic S clade adjacent to the genospecies *L. montravelensis* (Figure 2).

Whole-genome sequencing obtained from pathogenic *Leptospira* strains isolated from the two urine samples and all kidney samples had 100% identity with the deposited sequences in NCBI GenBank under BioProject accession number PRJNA731636 [36]. Furthermore, when the nucleotide sequence from environment strains isolated was compared with the other published genome sequences of *Leptospira*, we found a nucleotide similarity of 98.08% with the genomospecies *L. vanthielii* (NZ_RQHF00000000.1).

## 4. Discussion

The identification of the *Leptospira* species and genovariants circulating in Sardinian reservoir hosts is a fundamental prerequisite for the control and eradication of leptospirosis in this region. Recent studies in which pathogenic and intermediate strains of *Leptospira* have been isolated from clinical samples indicated that the disease is wide-spread among wildlife and domestic mammals on the island [16,17,18].

In this study, four pathogenic leptospiral strains all identified as *L. interrogans* serovar Pomona have been isolated from kidney organs of slaughtered swine in culture. These results confirm that Sardinia is not free from pathogenic *Leptospira* species and these findings establish the presence of this serovar in the study area, where this genospecies had been also isolated from wild (wild boar, fox, and dolphin) [16,37] and domestic animals (bovine and dogs) (data not published). However, this is the first identified outbreak caused by *L. interrogans* serovar Pomona in pigs from Sardinia.

These results agree with other studies in which it emerged that pigs are reservoir host species for the Pomona serotype, as well as for Bratislava and Tarassovi [38,39,40]. In this study, pigs from the infected farm were all asymptomatic. In general, endemic infections in swine herds remain subclinical, as do most leptospiral infections. Although clinical signs in these species are very difficult to observe and very limited clinical data are available thus far, the zoonotic potential of these strains has been already investigated [41]. When a susceptible herd becomes infected, considerable losses can occur due to abortion, stillbirths, weak piglets, or infertility [42,43,44,45]. Infections in pigs caused by other serotypes tend to occur only incidentally, vary regionally, and depend on other reservoir hosts, mainly rodents [46]. Leptospires colonize the porcine kidneys while serovar Bratislava colonizes mainly the genital tract, being excreted in urine and genital fluids [39].

In this study, the use of EMJH medium supplemented with 5-FU allowed the isolation and growth of new *Leptospira* serotype. Specifically, urine cultures were positives for saprophytic *Leptospira* compared with culture obtained from kidney samples as in this study. The two *Leptospira* strain isolates from urine cultures of one pig and one donkey were *L. montravelensis*, well-recognized as saprophytic *Leptospira*. To the best of our knowledge, except for two reports [47,48], there is no reliable information on isolation and molecular detection of saprophytic *Leptospira* species from clinical samples. In the present study, molecular analysis was able to confirm the total of positive samples obtained by MAT. The presence of *Leptospira* DNA has been successfully detected in urine and kidney samples using gene targets such as *rpoB* and *secY*, which detect the presence of all *Leptospira* species. However, isolation in culture does not allow the isolation of all samples with positive results following a molecular examination. Even if microbiological culture is the gold-standard method of diagnostic confirmatory for *Leptospira* identification [49], of eleven kidney samples (all from pigs) and six urine samples (three collected from pigs and three from donkeys) that tested PCR-positive for pathogen *Leptospira* spp., six had positive results after culture isolation. These data confirm that culture is less sensitive than molecular tests due to dead bacteria that cannot be cultured in tissue or at a lower level of detection. Furthermore, the fastidious nature of pathogenic leptospires makes their primary isolation and propagation from animal species a laborious and time-consuming task [49].

However, the number of saprophytic isolates (*n* = 2) was less than the number of the pathogenic ones (*n* = 4). All positive isolates were confirmed by molecular techniques using *Leptospira* genus primers that targeted a partial fragment of the *rrs* gene. In addition, since the diagnostic performance of this gene was lower in comparison with other target genes, two more sets of primers (*secY* and *rpoB* genes) were used to differentiate the sample isolated in a solid EMJH agar plate (in which no amplification for pathogenic leptospires was obtained). Our results suggested that the urine samples were co-infected with two different species belonging to the genus *Leptospira* (*L. interrogans* serovar Pomona and *Leptospira* sp. with 99–100% sequence similarities to *L. montravelensis*). We also hypothesize that fast-growing saprophytic microorganisms replaced the slow-growing pathogenic *Leptospira* in the enrichment medium, allowing these microorganisms to take over and establish themselves in the medium. As a future direction, further characterization of the isolates is needed to understand whether other saprophytic *Leptospira* species are included in positive molecular results.

Further analysis of the isolates obtained from the urine samples confirmed the presence of one saprophytic *Leptospira* species and WGS results suggested that these isolates were *L. montravelensis*. This finding is important, since this information has not been reported until now. However, the molecular presence of saprophytic *Leptospira* from rats has been reported [48], so this should also be considered. Although the molecular DNA investigation of the urine samples in this study amplified the target genes *rpoB*, *lipL32*, and *secY*, the MLST assay on the strain purified from urine cultures was negative. Our results confirm that these target genes and the loci used in the MLST assay are important tools for the identification of pathogenic *Leptospira* isolates, but are insufficient to define non-pathogenic *Leptospira* strains [50].

Saprophytic species are naturally present in environmental water and soil and do not usually cause disease [14]. Due to their non-pathogenicity, the presence of saprophytic *Leptospira* is not sought in clinical specimens, and the literature does not consider the zoonotic potential of saprophytic leptospires [51]. However, since previous studies have shown that both saprophytic and pathogenic leptospires are capable of forming biofilms [52], we can hypothesize that saprophytic leptospires (unlike the pathogenic ones that colonize the proximal renal tubules) can colonize the ureters, managing to survive the conditions of temperature and pH present in the urine. Since the urine samples were aseptically sampled from the bladder, this suggests that there was no contamination of the urine samples from the environment, which could occur with taking spontaneous urine (e.g., contaminated skin and vulva). We hypothesize that saprophytic *Leptospira* bacteria can survive and even multiply in the bladder or even—by retrograde migration—ascend in the ureter and maintaining here through biofilm. The early formation of biofilms by saprophytic strains of *Leptospira* compared to pathogenic species [53] is proposed as one of the mechanisms employed by leptospires to survive in environmental niches. The pathogenic species of *Leptospira* are probably less adapted to environmental conditions than the saprophytic species. Moreover, since a recent study showed that alive saprophytic leptospires enter and exit in both human and mouse macrophages with no intracellular replication [54,55], more studies are needed to understand the route of entry of saprophytic *Leptospira* into animal host. This might present a new insight into the transmission mechanisms of environmental saprophytic/intermediate strains of *Leptospira* through the urogenital route in pigs and donkeys that are exposed to contaminated soil and surface water. Future studies are needed to test the ability of *L. montravelensis* obtained from urine samples to form biofilms in ureters in vitro and to evaluate the pathogenic and epidemiological significance of these saprophytic strains, in a “One Health” approach [56].

## 5. Conclusions

Infection and close contact of farmers with infected pigs and donkeys could represent an important risk factor for the transmission of leptospirosis to humans. Carrying out regular screening of farm animals and households will enable conducting of active surveillance of leptospirosis to carry out risk assessments for prevention and treatment, and resource sharing to formulate a practical strategy for monitoring. Moreover, this study shows that pig urine samples harbor different *Leptospira* populations. Although saprophytic *Leptospira* are not considered a public health risk, future research should evaluate the range expansion of saprophytic *Leptospira* in sample hosts to expand the knowledge on this neglected group in new areas.

## Figures and Tables

**Table 1:** List of serum samples analyzed by MAT test and titers found.

Species	Sex	Titer Found
Pig 1	female	1:1600
Pig 2	female	1:1600
Pig 3	female	1:3200
Pig 4	female	1:3200
Pig 5	female	1:1600
Pig 6	female	1:800
Pig 7	female	1:3200
Pig 8	female	1:1600
Pig 9	female	1:1600
Pig 10	male	1:3200
Pig 11	male	1:1600
Donkey 1	male	1:800
Donkey 2	female	1:1600
Donkey 3	female	1:800

**Table 2:** Comparative results of molecular methods (multiplex qPCR1 and qPCR2 with cycle threshold (Ct) values) and cultural isolation of kidney and urine samples collected of pigs and donkeys in this study.

Samples	Matrix	qPCR1 (Ct Value)	qPCR2 (Ct Value)	Culture
Pig 1	kidney	Pos (35.8)	Neg	Neg
Pig 2	kidney	Pos (38.5)	Neg	Neg
Pig 3	kidney	Pos (24.0)	Neg	Pos
Pig 4	kidney	Pos (25.5)	Neg	Pos
Pig 5	kidney	Pos (32.2)	Neg	Pos
	urine	Pos (23.8)	Pos (15.1)	Pos
Pig 6	kidney	Pos (33.4)	Neg	Neg
Pig 7	kidney	Neg	Neg	Neg
Pig 8	kidney	Pos (22.7)	Neg	Pos
Pig 9	kidney	Neg	Neg	Neg
	urine	Pos (36.0)	Pos (26.8)	Neg
Pig 10	kidney	Neg	Neg	Neg
	urine	Pos (35.8)	Pos (21.5)	Neg
Pig 11	kidney	Pos (35.0)	Neg	Neg
Donkey 1	urine	Pos (37.4)	Neg	Neg
Donkey 2	urine	Pos (29.0)	Neg	Neg
Donkey 3	urine	Pos (29.2)	Neg	Pos

**Table 3:** Sequencing results of the *Leptospira* strains isolated from urine and kidneys samples.

Samples	Matrix	*rrs* Gene	*rpoB* Gene	*secY* Gene	MLST
Pig 3	kidney	*L. interrogans*	*L. interrogans*	*L. interrogans*	ST = 140
Pig 4	kidney	*L. interrogans*	*L. interrogans*	*L. interrogans*	ST = 140
Pig 5	kidney	*L. interrogans*	*L. interrogans*	*L. interrogans*	ST = 140
	urine	*L. interrogans*	*L. biflexa*	*L. biflexa*	Not determined
Pig 8	kidney	*L. interrogans*	*L. interrogans*	*L. interrogans*	ST = 140
Donkey 3	urine	*L. interrogans*	*L. biflexa*	*L. biflexa*	Not determined

## Data Availability

The Whole-Genome Shotgun project has been deposited at DDBJ/ENA/. GenBank accession number JANLJQ000000000 (BioProject accession number PRJNA867047, BioSample accession number SAMN30183847, and SRA accession number SRR22308640).
